# Dynamic Office Environments Improve Brain Activity and Attentional Performance Mediated by Increased Motor Activity

**DOI:** 10.3389/fnhum.2019.00121

**Published:** 2019-04-12

**Authors:** Diana Henz, Wolfgang I. Schöllhorn

**Affiliations:** Institute of Sports Science, Faculty of Social Sciences, Media and Sport, Johannes Gutenberg University Mainz, Mainz, Germany

**Keywords:** ergonomics, office, movement, dynamic working environment, EEG

## Abstract

Current research demonstrates beneficial effects of physical activity on brain functions and cognitive performance. To date, less is known on the effects of gross motor movements that do not fall into the category of sports-related aerobic or anaerobic exercise. In previous studies, we found beneficial effects of dynamic working environments, i.e., environments that encourage movements during cognitive task performance, on cognitive performance and corresponding brain activity. Aim of the present study was to examine the effects of working in a dynamic and a static office environment on attentional and vigilance performance, and on the corresponding electroencephalographic (EEG) brain oscillatory patterns. In a 2-week intervention study, participants worked either in a dynamic or a static office. In each intervention group, 12 subjects performed attentional and vigilance tasks. Spontaneous EEG was measured from 19 electrodes continuosly before, during, and immediately after each experimental condition at the first, and at the last intervention session. Results showed differences in EEG brain activity in the dynamic compared to the static office at the beginning as well as at the end of the intervention. EEG theta power increased in the vigilance task in anterior regions, alpha power in central and parietal regions in the dynamic compared to the static office. Further, increases in beta activity in the attention and vigilance task were shown in frontal and central regions in the dynamic office. Gamma power increased in the attention task in frontal and central regions. After 2 weeks, effects on brain activity increased in the attentional and vigilance task in the dynamic office. Increased theta and alpha oscillations were obtained in anterior areas with higher activity in the beta band in anterior and central areas in the dynamic compared to the static office. EEG oscillatory patterns indicate beneficial effects of dynamic office environments on attentional and vigilance performance that are mediated by increased motor activity. We discuss the obtained patterns of EEG oscillations in terms of the close interrelations between the attentional and the motor system.

## Introduction

Research demonstrates beneficial effects of bodily movement on brain and cognitive functions. Most studies performed in this area investigate the effects of aerobic exercise and gross motor movements on cognitive functions (for meta-analytic overviews see Etnier et al., 2004; Chang et al., 2012; Gheysen et al., 2018; Mandolesi et al., 2018). More specifically, beneficial effects of physical exercise have been demonstrated on brain and cognitive functions such as increased gray matter volume in frontal and hippocampal volume (Colcombe et al., 2006), increased neurotrophic factor (Brunoni et al., 2008), increased blood flow (Weinberg and Gould, 2015), increasing in academic achievement (Sibley and Etnier, 2003), improvements in cognitive functions such as memory abilities, efficiency of attentional and executive-control processes (Kramer et al., 1999; Colcombe and Kramer, 2003; Grego et al., 2005; Pereira et al., 2019; Winter et al., 2007; Chieffi et al., 2017), prevention of cognitive decline (Colberg et al., 2008), reduced risk developing dementia, and a modified network topology (Deeny et al., 2008). Examining the underlying neurophysiological processes, physical exercise induces most often changes in electroencephalographic (EEG) alpha and beta bands (Moraes et al., 2007, 2011). Further, changes in brain activity are dependent on the type and intensity of the physical exercise (Brümmer et al., 2011).

Less is known on the effects of gross motor movements on the cognitive system that do not fall into the categorization of aerobic or anaerobic exercise. In previous studies, we investigated the effects of dynamic sitting on brain activity and cognitive performance. Participants performed arithmetic, geometric, and algebraic tasks either on a static chair that did not foster movements or on a dynamic chair that allowed subject-induced movements in the vertical and horizontal direction during task performance. We observed improvements in mathematical performance when sitting on a dynamic chair. More specifically, performance in geometric and algebraic tasks that afforded visuo-spatial processing increased. The corresponding EEG brain activity showed increases in overall alpha and beta activity in areas related to visuo-spatial processing. We concluded that dynamic sitting activates working memory processes that leads to better performance in visuo-spatial processing (Henz, 2014; Henz et al., 2015).

These findings encourage further developments from dynamic sitting furniture towards the design of office environments consisting of mobile chairs, desks, and floors that foster motor activity which in turn stimulates the brain towards a state that is beneficial for cognitive task performance. These working environments consist of office desk furnitures that allows or even encourages movement behavior. In the applied research field of *neuroergonomics* findings from neuroscience and human factors are combined to design working environments that match the affordances and limitations of the human brain and cognitive system (for an overview see Ayaz and Dehais, 2019). One main aim is to detect the underlying brain and cognitive mechanisms that lead to a more efficient and healthy working. Research has focused on the effects of working environment designs on visual attention, vigilance, mental workload, working memory processes, and motor control on brain and cognitive functions (Parasuraman and Rizzo, 2007; Parasuraman and Wilson, 2008; Lees et al., 2010; Gramann et al., 2017). To date, no systematic studies have been performed on the effects of dynamic office furniture use on brain and cognitive functions in everyday working settings. These dynamic office furnitures have specific features that encourage movement behavior, i.e., chairs that allow vertical and horizontal movements during sitting, or desks that alter their height in an automated manner so that subjects have to adapt by altering their posture.

In the present study, we tested the effects of working in a dynamic office environment on EEG brain activity, attentional, and vigilance task performance. According to the results of previous studies (Henz, 2014; Henz et al., 2015), we expected that working in a dynamic office environment would improve attentional and vigilance performance. We hypothesized that working in a dynamic office environment would be accompanied by an increase in processing volume, and a decrease in errors in the attentional task. For the vigilance test, we expected a decrease in reaction times (RTs). Further, working in a dynamic office would activate the brain towards a state that fosters improvements in attentional and vigilance performance compared to working in a static office environment. We expected increases in beta and gamma power, and coherence in frontal electrodes in the attentional and vigilance task. Further, we hypothesized that improvements in attentional and vigilance performance accompanied by increases in beta and gamma power and increased coherence in the would increase after a 2-week intervention. For the EEG coherence analysis, we tested the frontal area, represented by the electrode pairs F3/F4, F3/Fz, and F7/Fz. EEG measured at these frontal electrode sites was assumed to reflect activity in (pre)frontal brain areas involved in executive functions, voluntary control, and action creation (Szurhaj et al., 2003; Moriguchi and Hiraki, 2013), Further, we tested the primary motor area from C3/Cz, C3/C4, and C4/Cz which is related to motor act execution (Toni et al., 2002), and the parietal area from P3/Pz, and P3/P4 that is involved in sensorimotor integration (Smith et al., 1999; Neuper and Pfurtscheller, 2001; Huber et al., 2006). Our hypotheses are based on findings of recent studies which have shown close interrelations between the motor system and attentional processing. Several investigations have shown increases in beta band activity when the motor network is activated (Khanna and Carmena, 2015; Chung et al., 2017). Beta activity is also eminent in brain areas that are related to attentional processing (Kopell et al., 2000). Synchronization in the beta range was shown in the dorsal prefrontal cortex and posterior parietal cortex in tasks that engage predominantly visual attention (Buschman et al., 2012; Verhoef et al., 2011). Saleh et al. (2010) demonstrated a close relation of beta activity in the motor cortex and beta activity during processing of attentional tasks.

## Materials and Methods

### Participants

Twenty-four subjects (mean age 24.3 years, age range 21–35, 12 males, 12 females) participated in the study. None of the subjects had current neurological diseases or a history of neurological impairments or intake of medication that may have influenced EEG brain activity. All subjects were right-handed. Handedness was assessed by the Edinburgh Handedness Inventory (Oldfield, 1971). Subjects were paid for participation in the study. The study was approved by the local ethics committee of the University of Mainz. All subjects gave written informed consent. All experimental procedures complied with the standards of the Helsinki Declaration of the World Medical Association Assembly. All subjects were naïve as to the purpose of the study.

### EEG Recording Details

EEG brain activity was recorded from 19 electrodes that were placed according to the international 10–20 system on the scalp with reference to the nose. EEG signals were recorded from the electrodes Fp1, Fp2, F3, F7, Fz, F4, F8, C3, Cz, C4, T3, T4, P3, P7, Pz, P4, P8, O1, O2. Electrodes are referred to different scalp areas as follows: frontal area (Fp1, Fp2, F3, F7, Fz, F4, F8), central area (C3, Cz, C4), temporal area (T3, T4), parietal area (P3, P7, Pz, P4, P8), and occipital area (O1, O2). The Micromed Brainquick amplifier (SD-LTM-32) and Micromed Brainspy software (Micromed, Venice, Italy) were used for the EEG recordings. Impedances of all electrodes were kept at 10 kΩ or below. EEG data were recorded continuously and digitized at a sampling rate of 256 Hz. EEG signals were amplified with a time constant of 0.3 s (high-pass filter: 0.5 Hz; low pass filter: 120 Hz; frequency range: 0.5–120 Hz). To assess electrooculographic (EOG) data two electrodes were placed at the medial upper and lateral orbital rim of the right eye. EOG signals were amplified with a time constant of 0.3 s (high pass filter: 0.1 Hz; low pass filter: 120 Hz; frequency range: 0.5–120 Hz). Heart rate was measured continuously as a control variable using the medilog^®^ AR12plus recorder (Schiller, Linz, Austria) at a sampling rate of 1,000 Hz. As a further control variable, electromyographic (EMG) activity was recorded using the biovision (Wehrheim, Germany) from two electrodes placed at the splenius capitis, and two electrodes placed at the trapezius pars descendens at a sampling rate of 500 Hz.

### Dynamic and Static Office Environments

**Figure 1:**
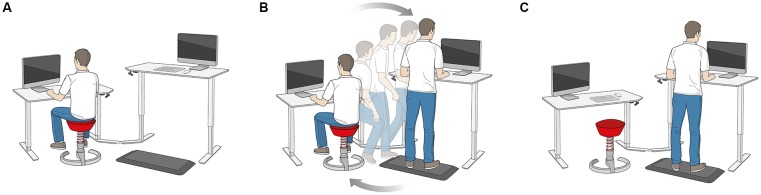
Illustration of the dynamic office environment. **(A)** Sitting workstation. **(B)** Change from the sitting to the standing workstation. **(C)** Standing workstation.

A schematic illustration of the dynamic office environment (active office^®^, aeris GmbH, Munich, Germany) is depicted in Figure 1. It consists of two desks that are height-adjustable with one desk designed as a sitting workstation, and the other as a standing workstation. Subjects sat at the sitting workstation on a height-adjustable stool (swopper^®^, aeris GmbH, Munich, Germany) that allowed subject-induced movements in the vertical direction. At the standing workstation, subjects performed tasks placed on a height-adjustable stool (muvman^®^, aeris GmbH, Munich, Germany) that supports sitting with legs stretched similar to a stance position. Additionally, subjects stood on a foam mat that had an uneven surface structure (aeris^®^ muvmat) at the standing workstation. A 21″ screen was placed on the middle of each desk. One main characteristic of the dynamic office environment is that subjects have to change the workstation at randomly set time intervals ranging from 5 to 20 min. The experimental tasks were arranged in that way that after completion of some sections a signal on the screen appeared that indicated to change the workstation. In contrast, subjects performed the experimental tasks in the static office environment in the same desk configuration but sat only at the sitting workstation on a static stool. The static stool was height-adjustable and had the same properties as the stool used in the dynamic condition except of allowing subject-induced movements in the vertical direction.

### Assessment of Attentional and Vigilance Performance

**Figure 2:**
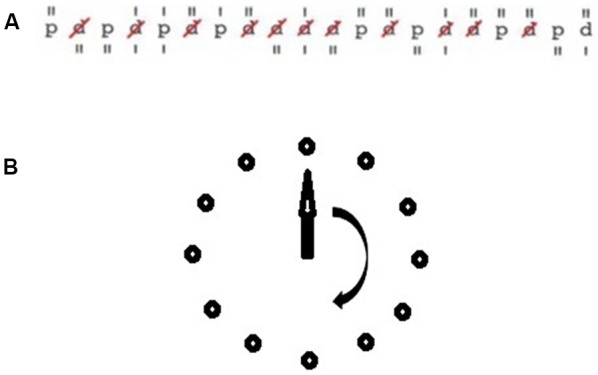
Illustration of the experimental tasks. **(A)** Sample row of the d2-R task. **(B)** Mackworth clock task.

The experimental tasks are illustrated in Figure 2. For the assessment of short-term attentional performance, subjects performed the d2-R attention test (Brickenkamp et al., 2010). The test sheet of the d2-R test consists of 14 lines with 57 characters each. The characters are the letters “d” and “p”. They are accompanied by different markers consisting of one to four vertical lines that are positioned over or below the characters. The task of the subjects was to scratch all letters “d” with a total of two lines. Errors consisted of missing to scratch a letter “d”, the scratching of the letter “p”, and the scratching of a letter “d” with more or less than a total of two lines. The degree of difficulty of the test is composed of the time limit (4 min 40 s) and the challenge to discriminate between relevant and irrelevant stimuli. Vigilance performance was assessed by the Mackworth clock task (Mackworth, 1948). Test stimuli were presented on a screen. Subjects tracked a clock hand visually that moved around the screen passing distinct positions that were arranged circular (diameter 15 cm). The task of the subjects was to press a button when the hand jumped more than one position. RTs are calculated from the latency of the hand jump, and the button press. RTs were taken for the segments: (1) minute 1–5; (2) minute 6–10; (3) minute 11–15; and (4) minute 16–20.

### Experimental Procedure

Prior to the experiment, the experimental tasks were explained. Each subject was shown where and how to sit or stand. Half of the subjects performed the intervention in the dynamic office environment, the other half underwent the intervention in the static office environment. Participants were randomly assigned to the experimental groups. After subjects gave their informed consent, demographic data were assessed. Then, they began with a 5-min resting condition. Spontaneous EEG was recorded for 5 min with eyes-open. Then, subjects were sat at the office environment and performed the experimental tasks. Each intervention session had a duration of 4 h. Subjects performed a standardized program that contained everyday office tasks (E-mail correspondence, calculations, document reading etc.). The d2-R test was performed after 10 min and after 120 min, the Mackworth clock task after 15 min and after 125 min working in the office in the first and in the last session of the 2-week intervention. Spontaneous EEG was recorded continuously during the six conditions: (1) pre-intervention rest; (2) d2-R test; (3) Mackworth clock task minutes 1–5; (4) Mackworth clock task minutes 6–10; (5) Mackworth clock task minutes 11–15; and (6) Mackworth clock task minutes 16–20 that were used for subsequent statistical analyses.

### EEG Analysis

EEG analyses were performed with the EEGLAB (Swartz Center for Computational Neuroscience, La Jolla, CA, USA). Spontaneous EEG was assessed with eyes-open. Five-minute sequences were recorded before and after each experimental condition. Independent component analyses (ICAs) were performed for the EEG signal. Components that resulted from artifacts were removed. For the analysis of the EEG data, Fast Fourier Transforms were performed to calculate the mean power spectra for the theta (4–7.5 Hz), alpha (8–13 Hz), beta (14–30 Hz), and gamma (31–70 Hz) bands. Further, EEG coherence for the electrode pairs (F3/F4, F3/Fz, F7/Fz, C3/Cz, P3/Pz, and P3/P4) was analyzed. Coherence was calculated for the theta, alpha, beta, and gamma range.

### Electrode Spatial Localization

Three regions of interest were selected: the pre-motor and pre-frontal cortex, represented by the F3/F4, F3/Fz and F7/Fz electrode pairs, which are assumed to be functionally involved in action creation and voluntary control (Moriguchi and Hiraki, 2013), and for executive functions (Szurhaj et al., 2003). The C3/Cz, C3/C4 and C4/Cz electrode pairs were assessed for being representatives of primary motor areas related to the motor act execution (Toni et al., 2002); the P3/Pz and P3/P4 electrode pairs due to their relation to sensorimotor integration (Smith et al., 1999; Huber et al., 2006).

### Data on the Attention and Vigilance Task

Performance in the d2-R test was determined by the attention performance score. It was calculated by subtracting the number of falsely marked test items from the total number of correctly marked characters. Mean RTs in the Mackworth clock task were calculated for four segments: (1) minute 1–5; (2) minute 6–10; (3) minute 11–15; and (4) minute 16–20.

### Statistical Analyses

Means and standard deviations of the d2-R attentional performance score, and the RTs of the Mackworth clock task (minute 1–5, minute 6–10, minute 11–15, minute 16–20) were calculated. Mauchly’s test of sphericity was calculated to test the assumptions of repeated-measures analysis of variance (ANOVAs) for the d2-R test score, the RTs of the Mackworth clock task, and the EEG data. Consecutive ANOVAs were performed when the *p*-values were equal or exceeded 0.05. A two-way ANOVA that included the between-subjects factor working environment (static, dynamic), and the within-subjects factor time (pretest, posttest) was performed for the d2-R test score. Further, a three-way ANOVA that included the between-subjects factor working environment (static, dynamic), and the within-subjects factors time (pretest, posttest), and segment (minute 1–5, minute 6–10, minute 11–15, minute 16–20) was performed for the RTs of the Mackworth clock task. In a consecutive step, data were subjected to *post hoc* *t*-tests with Bonferroni–correction. For the EEG data, repeated-measure ANOVAs were performed separately for the theta, alpha, beta, and gamma bands that included the between-subject factor working environment (static, dynamic), and the within-subject factors as time (pretest, posttest), experimental condition (pretest rest, vigilance test minute 1–5, vigilance test minute 6–10, vigilance test minute 11–15, vigilance test minute 16–20, posttest rest), and location (Frontal, Central, Temporal, Parietal, Occipital). In a consecutive step, *post hoc* *t*-tests with Bonferroni-correction were calculated for significant main or interaction effects. Additionally, partial eta-squared ($\eta_{\text{p}}^{2}$) was calculated to determine effect sizes for the d2-R test score, RTs of the Mackworth clock task, and the frequency specific EEG power densities (theta, alpha, beta, gamma). The coherence values were analyzed using a two-way ANOVA with the factors working environment (static, dynamic), and time (pretest, posttest) for each electrode pair studied (F3/F4, F3/Fz, F7/Fz, C3/Cz, P3/Pz, P3/P4 and T3/T4). Statistical significance of the tests was achieved when the *p*-values were less than 0.05.

## Results

### Attentional Performance

**Figure 3:**
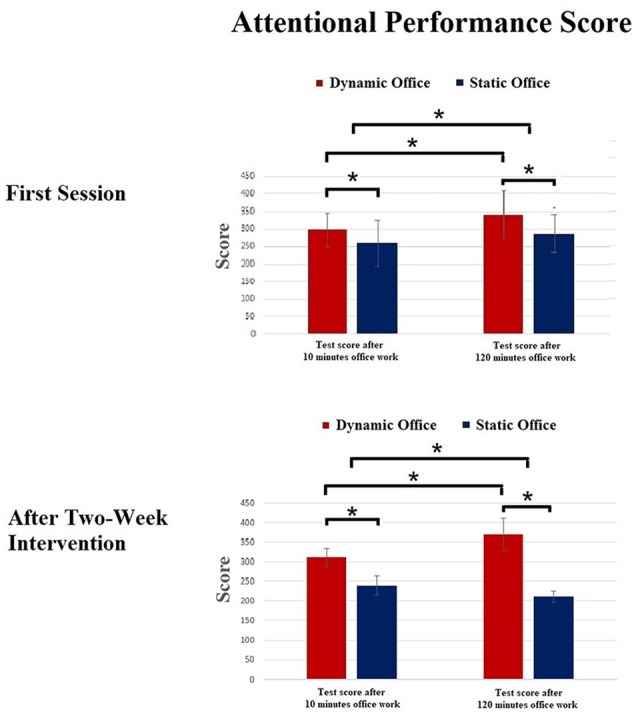
Results of the d2-R test. *p < 0.05.

**Figure 4:**
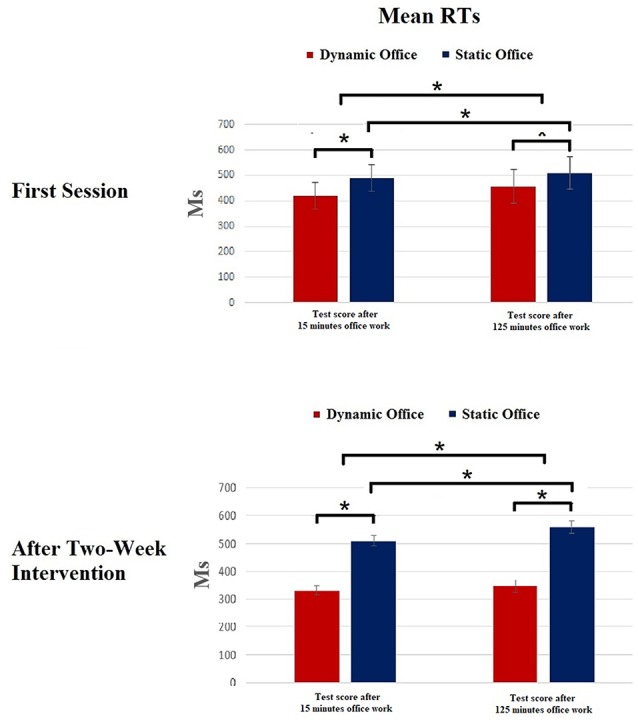
Results of the Mackworth clock task. *p < 0.05.

Means and standard deviations of the scores of the d2-R test and the Mackworth clock task are depicted in Figures 3, 4. The ANOVA on the attentional performance score of the d2-R test showed a significant effect for the factor working environment after 10 min, *F*_(1,23)_ = 5.92, *p* = 0.02, $\eta_{\text{p}}^{2}$ = 0.18. Further, the effect for the factor time was significant, *F*_(1,19)_ = 7.39, *p* = 0.02, $\eta_{\text{p}}^{2}$ = 0.21. The working environment × time interaction was significant, *F*_(4,19)_ = 3.14, *p* = 0.03, $\eta_{\text{p}}^{2}$ = 0.13.

The ANOVA for the RTs in the Mackworth clock task revealed a significant effect for working environment, *F*_(1,19)_ = 4.75, *p* = 0.03, $\eta_{\text{p}}^{2}$ = 0.15. Further, a significant main effect was shown for the factors experimental condition, *F*_(3,57)_ = 3.81, *p* = 0.02, $\eta_{\text{p}}^{2}$ = 0.11, and time, *F*_(1,19)_ = 6.03, *p* = 0.02, $\eta_{\text{p}}^{2}$ = 0.17. The working environment × time interaction was significant, *F*_(4,19)_ = 3.77, *p* = 0.04, $\eta_{\text{p}}^{2}$ = 0.06.

### Spontaneous EEG

**Figure 5:**
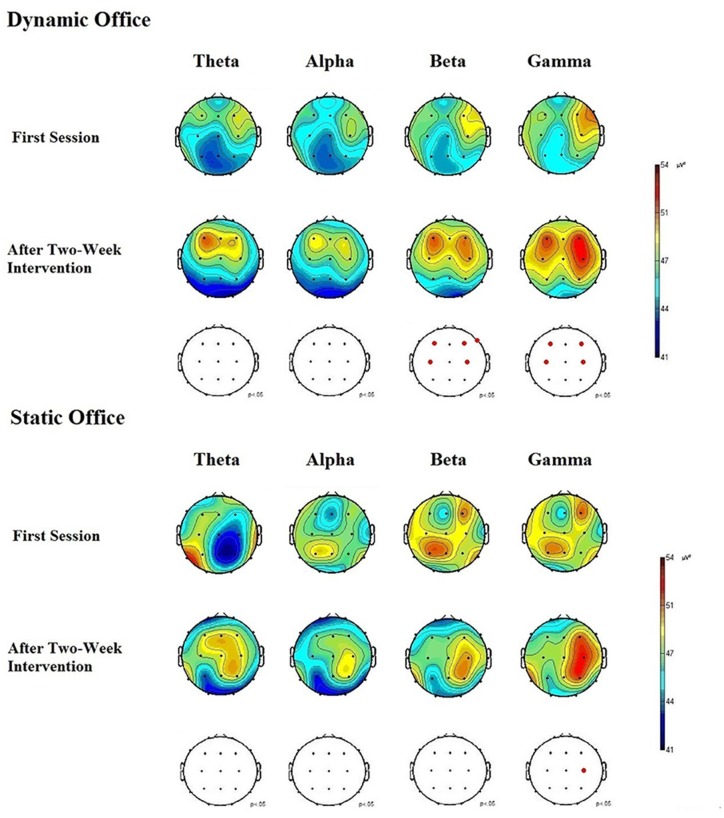
Electroencephalographic (EEG) brain activity for the theta, alpha, beta, and gamma bands in the dynamic and static office during the d2-R test.

**Figure 6:**
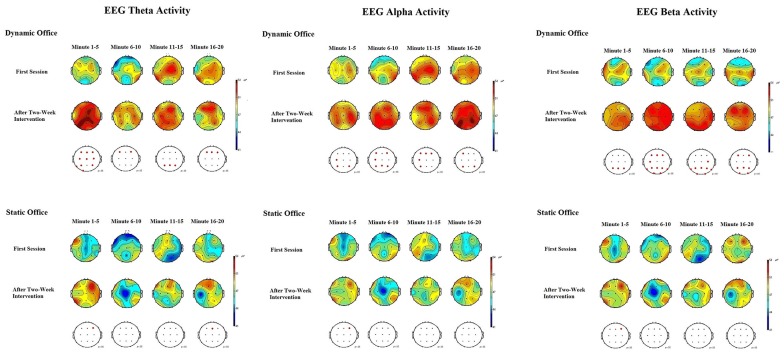
EEG brain activity for the theta, alpha, and beta bands in the dynamic and static office during the Mackworth clock task.

Mean power spectra for the EEG theta, alpha, beta, and gamma bands in the d2-R test and the Mackworth clock task are depicted in Figures 5, 6.

The ANOVA for theta power obtained during the Mackworth clock task showed significant main effects for working environment, *F*_(1,19)_ = 4.92, *p* = 0.03, $\eta_{\text{p}}^{2}$ = 0.08, and time, *F*_(1,19)_ = 4.53, *p* = 0.04, $\eta_{\text{p}}^{2}$ = 0.05. The working environment × time interaction was significant, *F*_(4,19)_ = 2.96, *p* = 0.04, $\eta_{\text{p}}^{2}$ = 0.03. A further ANOVA revealed significant differences between locations, *F*_(20,95)_ = 2.16, *p* = 0.02, $\eta_{\text{p}}^{2}$ = 0.13. *Post hoc* comparisons showed increases in EEG theta power at frontal, central, and parietal electrodes, compared to temporal and occipital electrodes, *p* < 0.05 each. Subsequent analyses showed that theta power increased most in the frontal region at the electrodes F3 and F4, *p* < 0.05 each.

The ANOVA for alpha power obtained during the Mackworth clock task showed a significant main effects for working environment, *F*_(1,19)_ = 5.96, *p* = 0.02, $\eta_{\text{p}}^{2}$ = 0.14, and time, *F*_(1,19)_ = 6.16, *p* = 0.02, $\eta_{\text{p}}^{2}$ = 0.15. The working environment × time interaction was significant, *F*_(4,19)_ = 3.02, *p* = 0.03, $\eta_{\text{p}}^{2}$ = 0.12. The ANOVA of alpha responses revealed significant differences between locations, *F*_(20,95)_ = 1.90, *p* = 0.04, $\eta_{\text{p}}^{2}$ = 0.05. *Post hoc* comparisons showed that spontaneous EEG alpha power was higher at central and parietal electrodes, than that of frontal, temporal, and occipital electrodes, *p* < 0.05 each. Subsequent analyses showed that alpha power increased most in the central region at the electrode C4, and in the parietal region at the electrodes P3 and P4, *p* < 0.05 each.

The ANOVA for beta power obtained during the d2-R test showed highly significant main effects for the factors working environment, *F*_(1,19)_ = 8.45, *p* = 0.01, $\eta_{\text{p}}^{2}$ = 0.25, and time, *F*_(1,19)_ = 8.37, *p* = 0.01, $\eta_{\text{p}}^{2}$ = 0.22. The working environment × time interaction was significant, *F*_(4,19)_ = 3.84, *p* = 0.02, $\eta_{\text{p}}^{2}$ = 0.18. The ANOVA of beta responses revealed highly significant differences between locations, *F*_(20,95)_ = 2.83, *p* = 0.009, $\eta_{\text{p}}^{2}$ = 0.28. *Post hoc* comparisons showed that spontaneous EEG beta power was higher at frontal and central electrodes, than that of parietal, temporal, and occipital electrodes, *p* < 0.05 each. Subsequent analyses showed that beta power increased most in the frontal region at the electrodes F3, F4, and F8, *p* < 0.01 each, and in the central region at the electrodes C3 and C4, *p* < 0.05 each. The ANOVA for beta activity obtained during the Mackworth clock task showed highly significant main effects for the factors working environment, *F*_(1,19)_ = 8.48, *p* = 0.01, $\eta_{\text{p}}^{2}$ = 0.27, and time, *F*_(1,19)_ = 8.25, *p* = 0.01, $\eta_{\text{p}}^{2}$ = 0.24. The working environment × time interaction was significant, *F*_(5,95)_ = 3.28, *p* = 0.03, $\eta_{\text{p}}^{2}$ = 0.17.

The ANOVA for gamma power obtained during the d2-R test showed significant main effects for working environment, *F*_(1,19)_ = 5.17, *p* = 0.02, $\eta_{\text{p}}^{2}$ = 0.10, and time, *F*_(1,19)_ = 5.03, *p* = 0.03, $\eta_{\text{p}}^{2}$ = 0.08. The working environment × time interaction was significant, *F*_(4, 19)_ = 3.38, *p* = 0.03, $\eta_{\text{p}}^{2}$ = 0.10. The ANOVA of gamma responses revealed a highly significant difference between locations, *F*_(20,95)_ = 2.79, *p* = 0.008, $\eta_{\text{p}}^{2}$ = 0.28. *Post hoc* comparisons showed that spontaneous EEG gamma power increased at frontal and central electrodes, compared to that of parietal, temporal, and occipital electrodes, *p* < 0.05 each. Subsequent analyses showed that gamma power increased most in the frontal region at the electrodes F3 and F4, *p* < 0.05, and in the central region at the electrodes C3 and C4, *p* < 0.05 each.

### EEG Coherence

For the theta range, highly significant main effects were found in the Mackworth clock task for the electrode pairs F3/F4, *F*_(1,19)_ = 10.71, *p* = 0.01, and P3/P4, *F*_(1,19)_ = 14.82, *p* = 0.008, with a coherence increase in the dynamic office, when compared to the static office. Further, highly significant main effects were found for the factor time for the electrode pairs F3/F4, *F*_(1,19)_ = 9.75, *p* = 0.01, and P3/P4, *F*_(1,19)_ = 10.48, *p* = 0.009, with increases in theta coherence in the posttest, when compared to pretest. The working environment × time interactions were significant for F3/F4, *F*_(4,19)_ = 3.27, *p* = 0.02, and P3/P4, *F*_(4,19)_ = 4.22, *p* = 0.02.

Analysis on the alpha range showed significant main effects in the Mackworth clock task for the electrode pairs F3/F4, *F*_(1,19)_ = 5.62, *p* = 0.03, and P3/P4, *F*_(1,19)_ = 6.81, *p* = 0.02, with coherence increases in the dynamic office, when compared to the static office. Further, significant main effects were found for the factor time for the electrode pairs F3/F4, *F*_(1,19)_ = 4.88, *p* = 0.04, and P3/P4, *F*_(1,19)_ = 5.74, *p* = 0.03, with increases in alpha coherence in the posttest, when compared to pretest. The working environment × time interactions were significant for F3/F4, *F*_(4,19)_ = 4.02, *p* = 0.02, and P3/P4, *F*_(4,19)_ = 5.82, *p* = 0.03.

For the beta range, significant main effects were found in the Mackworth clock task for the electrode pairs F3/F4, *F*_(1,19)_ = 8.02, *p* = 0.02, and C3/C4, *F*_(1,19)_ = 7.87, *p* = 0.02, with a coherence increase in the dynamic office, when compared to the static office. Further, main effects were found for the factor time for the electrode pairs F3/F4, *F*_(1,19)_ = 7.53, *p* = 0.02, and C3/C4, *F*_(1,19)_ = 7.96, *p* = 0.02, with increases in beta coherence in the posttest, when compared to pretest. The working environment × time interactions were significant for F3/F4, *F*_(4,19)_ = 3.90, *p* = 0.03, and C3/C4, *F*_(1,19)_ = 4.05, *p* = 0.02. Further for the d2-R task, highly significant main effects were found for the electrode pairs F3/F4, *F*_(1,19)_ = 10.57, *p* = 0.009, and C3/C4, *F*_(1,19)_ = 8.73, *p* = 0.01, with a coherence increase in the dynamic office, when compared to the static office. Further, significant main effects were found for the factor time for the electrode pairs F3/F4, *F*_(1,19)_ = 7.80, *p* = 0.02, and C3/C4, *F*_(1,19)_ = 6.33, *p* = 0.02, with increases in beta coherence in the posttest, when compared to pretest. The working environment × time interactions were significant for F3/F4, *F*_(4,19)_ = 3.98, *p* = 0.03, and C3/C4, *F*_(4,19)_ = 3.25, *p* = 0.03.

Analysis on the gamma range showed highly significant main effects in the d2-R task for the electrode pairs F3/F4, *F*_(1,19)_ = 10.23, *p* = 0.009, and P3/P4, *F*_(1,19)_ = 9.27, *p* = 0.01, with coherence increases in the dynamic office, when compared to the static office. Further, a highly significant main effects was found for the factor time for the electrode pairs F3/F4, *F*_(1,19)_ = 11.06, *p* = 0.008, and P3/P4, *F*_(1,19)_ = 8.97, *p* = 0.01, with increases in gamma coherence in the posttest, when compared to pretest. The working environment × time interactions were significant for F3/F4, *F*_(4,19)_ = 5.38, *p* = 0.01, and P3/P4, *F*_(4,19)_ = 3.86, *p* = 0.02.

## Discussion

This is the first study that investigated the effects of a dynamic office environment on EEG brain oscillations and attentional as well as vigilance performance. Results clearly demonstrate distinguishable patterns of EEG brain oscillations when working in the dynamic compared to working in the static office. Attentional performance increased in the dynamic office compared to working in the static office after 120 min. Moreover, attentional and vigilance performance increased after the 2-week intervention in the dynamic office. Brain oscillatory patterns showed increased beta and gamma power in frontal (F4), and parietal (P3, Pz) areas in the attentional task in the dynamic office. Theta, alpha, and beta power increased in frontal (F3, Fz, F4), central (C3), and parietal (P3, Pz, P4) areas in the vigilance task when working in the dynamic office. Further, analyses revealed increased interhemispheric coherence for the electrode pairs F3/F4, C3/C4, and P3/P4 in the dynamic office. Effects on brain oscillatory patterns increased after the 2-week intervention in the dynamic office. We found increased EEG alpha, beta, and gamma power in the d2-R task as compared to pretest. Further, EEG theta, alpha, and beta power were higher in the vigilance task than at pretest. EEG data on coherence demonstrated increased interhemispheric coherence for the electrode pairs F3/F4, C3/C4, and P3/P4 at posttest.

Our results expand findings from previous neurophysiological investigations on the beneficial effects of dynamic sitting on cognitive functions and brain oscillations (Henz and Schöllhorn, 2016; Henz et al., 2015). In these studies, we showed increases in visuo-spatial and working memory task performance when sitting on a chair that allowed movements during working compared to working on a static chair. As a neural correlate for improved task performance, we found increases in alpha and beta power in brain areas that are related to visuo-spatial and working memory processes. We argued that these improvements are mediated by increased motor activity in the dynamic sitting condition.

The obtained patterns of EEG brain oscillations indicate different underlying neural processes during working in a dynamic office versus working in a static office environment. In the following sections, we discuss different lines of interpretations on the obtained patterns of brain oscillations.

### Dynamic Office Environments Enhance Brain Oscillations Related to Attentional Processing

Brain oscillations before and after the 2-week intervention in the dynamic office showed increases in frontal (F3, F4), central (C3), and parietal (P3, P4) theta, alpha, and beta band activity during the vigilance task compared to pretest. Coherence analysis showed increased interhemispheric coherence for the derivations F3/F4, C3/C4, and P3/P4. We argue that the observed brain oscillatory patterns are a correlate for modulations of attentional processes during working in the dynamic office. Previous studies have demonstrated that frontal cortexes are related to executive functions, voluntary control, and action creation (Jung et al., 2000; Cardoso de Oliveira, 2002). Theta activity was shown to be related to these behaviors that demand action planning based on received sensory information (Liepert et al., 1998; Caplan et al., 2003). These studies show a relation between theta band coherence with attention and movement preparation.

Results on increased EEG theta power during the Mackworth clock task are in line with findings of a study by Pennekamp et al. (1994). They showed increases in performance in the Mackworth clock task that were accompanied by increases in theta activity. Several studies have shown that the allocation of attentional resources is accompanied by distinct brain activation patterns. Enhanced theta activity in frontal brain areas is currently discussed as a neural substrate for improvements of cognitive control (Chung et al., 2017). More specifically, theta oscillations in frontal brain areas are observed in the early allocation of selective attention resources on external visual stimuli. This frontal theta activity appears to improve cognitive control during visuo-motor tasks (Berchicci et al., 2015). Increases in theta activity are shown in goal-directed attention (Dowdall et al., 2012). Stimulus-induced changes in the theta, alpha, and beta bands have been shown to be a correlate for modulation of goal-directed spatial attention (Harris et al., 2017). More specifically, EEG oscillations occurred either in the theta, alpha, and beta range in the involuntary capture of goal-directed visual attention. The authors found a lateralization in the theta band related to the processing of goal-relevant and goal-irrelevant stimuli. Lateralization in the alpha band was shown for goal-directed attention. Further, beta oscillations were not location-specific. Several studies have shown that alpha activity in parietal regions is involved in the processing of visual stimuli (Jensen et al., 2012; Roux and Uhlhaas, 2014; Foster et al., 2016). Further, there is evidence for a relation between alpha oscillations and voluntary attentional allocation (Worden et al., 2000; Kelly et al., 2006; Thut et al., 2006; Foxe and Snyder, 2011).

### Relations Between Increased Motor Activity and Attention

Attentional and vigilance performance increased in the dynamic compared to the static office. Analysis on the corresponding power spectra and coherence analysis revealed increased activity in the frontal areas that are related to executive functions, voluntary control, and action planning (Szurhaj et al., 2003; Moriguchi and Hiraki, 2013). Further, brain areas related to motor act execution showed increased oscillatory activity. Increased coherence in C3/C4 is associated with motor act execution (Papenberg et al., 2013), motor act organization (Minc et al., 2010), and sequential movement coordination (Coull et al., 1998). Such findings show that motor activity and executive processes are closely related.

Current research shows close interrelations between attentional processing and posture control. Recent studies using a dual-task paradigm indicate that sensorimotor processing is essential to postural control, and requires attentional resources (Boisgontier et al., 2013). Several investigations reveal that even highly practiced postural tasks require cognitive processing at least to a small degree. For instance, Tsang et al. (2016) have shown that the simultaneous performance of a cognitive task during quiet stance resulted in an increase in postural sway. Beta activity has been shown in attention and long-distance synchronization in parts of the cortex. For instance, synchronization of beta activity was demonstrated in the dorsal prefrontal cortex and posterior parietal cortex during a top-down search of a visual stimulus (Buschman and Miller, 2007; Buschman et al., 2012; Verhoef et al., 2011). Beta activation in motor areas is a neural substrate for attention to upcoming motor tasks. Brain activation patterns in the beta range are not limited to the activity of the motor cortex and EMG, but play also an important role in somatosensory and parietal areas (Witham and Baker, 2007; Tsujimoto et al., 2009). Further, coherence between the motor and afferent sensory areas is shown to operate at the beta frequency (Baker, 2007). Saleh et al. (2010) demonstrated a close link between beta activity in the motor cortex and attention. Further, beta activity indicates selectively coordinated cell-assemblies that are task-relevant (Kopell et al., 2000). EEG beta activity also plays an important role in attentional processing (Wróbel et al., 2007; Sauseng and Klimesch, 2008). Recent studies suggest that EEG beta activity is necessary to enhance feedback loops at subsequent stages of visual information processing (i.e., Gola et al., 2013). Beta activity and connectivity in the sensorimotor and parietal cortex are essential for accurate motor performance (Chung et al., 2017).

A further line of argumentation considers the role of alpha and beta activation in self-initiated movements. Wang et al. (2017) found increases in alpha activity in the SMA in self-initiated movements. Further, they demonstrated increases in sensorimotor areas in the alpha and beta bands in self-initiated and visually guided movements.

Finally, we found increases in gamma power during the attentional task when working in the dynamic office. We interpret these results in terms of an enhancing effect of motor activity on attentional processing. Increases in gamma oscillations are found in selective attentional processing (Engel and Singer, 2001; Varela et al., 2001). From this, we argue that performing attentional tasks in a dynamic office environment stimulates executive cognitive controlled processing. This interpretation is in line with studies that showed enhancement of executive functions by gross motor movements (i.e., Benzinger et al., 2018). Further, evidence shows that gamma band activity of frontal and central areas is associated with memory encoding (Sederberg et al., 2003), motor memory tasks (Gentili et al., 2015), and encoding of motor memory in dynamic motor adaptation tasks (Thürer et al., 2016).

In summary, several studies have shown that theta, alpha, and beta frequencies play dissociable roles in visual attention. Increases in gamma power were found as a correlate for selective attention. From this, we interpret the increases in the theta, alpha, and beta oscillations in the vigilance task, and the increases in the alpha, beta, and gamma bands in the attentional task as indicators for modulations of visual attention that result from increased motor activity when working in a dynamic office that fosters physical activity.

### Working in a Dynamic Environment Reinforces Visuo-Spatial Working Memory Processes and Multisensory Integration

Increases in theta band oscillations have also been discussed as an indicator for visuo-spatial working memory processes (Bastiaansen et al., 2002). Further, resource allocation is one of the cognitive processes that are located within working memory models. Increases in theta power in somatosensory and motor brain areas during working in the dynamic office might be a correlate for working memory processes (Carretié, 2001; Mitchell et al., 2008; Myers et al., 2014; Tóth et al., 2014), and a neurophysiological substrate for encoding of new information (Klimesch et al., 1996, 1997; Klimesch, 1999; Bastiaansen et al., 2002). In previous studies, we found enhancement of visuo-spatial abilities in geometrical and algebraic tasks during sitting on a chair that allowed vertical and horizontal movements during sitting. Enhanced performance was accompanied by increases in frontal theta activity (Henz, 2014; Henz et al., 2015). We argued that better performance in geometrical and algebraic tasks resulted from stimulation of the visuo-spatial working memory system by bodily movements.

A further line of interpretation is that increases in theta and alpha coherence reflect processes of multisensory integration in the brain that afford working memory processes (see Kanayama et al., 2015). For instance, Hummel and Gerloff (2005) demonstrated increases in alpha coherence during a cross-modal matching task. The authors argue that the EEG alpha coherence is a correlate for synchronization processes of brain areas that are related to cross-modal integration. Classen et al. (1998) showed increased EEG coherence between visual and somatosensory brain areas as well as between visual and motor areas during a visuo-motor tracking task. Further studies have shown increased theta coherence in the derivations P3/Pz, and P3/P4 which are involved in sensorimotor integration (Smith et al., 1999; Neuper and Pfurtscheller, 2001; Huber et al., 2006). Several studies show that processing of whole-body movements is accompanied by modulations in alpha band activity. Gutteling and Medendorp (2016) demonstrated that the processing of bodily motion is mirrored by a modulation in central and parietal areas, whereas visual target coding was shown in power modulation in parieto-occipital areas. Summarizing, the parietal cortex is involved in both the processing of bodily motion, and spatial processing, consistent with its role as a brain region that integrates information from visual, motor and vestibular signals (Zhang and Britten, 2011; Gale et al., 2015; Gutteling et al., 2015).

### Increased Fluctuations Stimulate Brain Functions and Attentional Performance

A different theoretical perspective on the effects of increased motor activity on brain oscillatory patterns and attentional performance could be applied considering theoretical assumptions of the system dynamic theory (Haken, 1970; Glansdorff and Prigogine, 1971; Haken et al., 1985). Bodily movements that change continuously during task performance and problem solving might lead to increased fluctuations and deviations during task solving. Several scientific approaches refer to the term *deviation* from a predefined ideal state. From this perspective, the term deviation has a rather negative connotation. In contrast, the system dynamic approach considers deviations rather as constructive fluctuations. They are defined as a more neutral term that is derived from stochastic physics. According to the system dynamic approach, systems that are characterized to have energetic or material exchange with an environment show continuous fluctuations. Another hypothesis derived from the system dynamic approach is that an increase of fluctuations that causes a period of instability is necessary to stimulate transitions from one stable state to another state in these systems. During phase transitions, these systems explore a variety of modes in order to find new and even more effective states. These phenomena have been investigated extensively in different areas of sports and everyday movements (Kelso, 1995; Davids et al., 2006). Recent neurophysiological studies have shown that increased fluctuations in the human body induced by differential movement training increased learning rates that were accompanied by brain oscillations in the theta and alpha range (Henz and Schöllhorn, 2016; Henz et al., 2018). Instead of considering increases in fluctuations as a passive ontological phenomenon of dissipative systems, the differential training approach takes advantage of increased system fluctuations as an active instrument in order to lead the system towards a zone of instability where less energy is needed for achieving a self-organized new state (Schöllhorn, 1999, 2000; Schöllhorn et al., 2006, 2009, 2010, 2012). One implication of these previous findings is that increased fluctuations in the human body induced by randomly performed movements enhance a state of brain oscillatory activity that is characterized by increased theta and alpha power which reinforces creative solutions in sports (Santos et al., 2018) as well as during problem solving (Fink et al., 2007, 2009) in working and school settings.

## Conclusion

The results of the present study reveal short- and mid-term effects on attentional and vigilance performance, and EEG brain activity when working in a dynamic versus a static environment. During working in a dynamic office, attentional and vigilance performance increased compared to working in a static office. Brain activities show increased alpha, beta and gamma power in the frontal and central areas in the attentional task with increased theta, alpha, and beta activity in the vigilance task. These findings suggest that working in a dynamic office environment stimulates the brain towards an optimum psychophysiological level of activation and wakefulness for attentional and vigilance performance. The results of the present study are of relevance in the field of neuroergonomics, for the design of office and school working environments and encourage the use of dynamic office desk furniture that allows movements during working to achieve increased attentional as well as vigilance performance.

## Ethics Statement

The study was approved by the local ethics committee of the University of Mainz. All subjects gave written informed consent. All experimental procedures complied with the standards of the Helsinki Declaration of the World Medical Association Assembly. All subjects were naïve as to the purpose of the study.

## Author Contributions

All listed authors made substantial and intellectual contributions to the manuscript.

## Conflict of Interest Statement

The authors declare that the research was conducted in the absence of any commercial or financial relationships that could be construed as a potential conflict of interest.
